# Anxiety modulates odour-linked brain connectivity and alcohol dependence risk

**DOI:** 10.1093/braincomms/fcag096

**Published:** 2026-03-19

**Authors:** Khushbu Agarwal, Shefali Chaudhary, Valentina Parma, Yatan Pal Singh Balhara, Siddharth Sarkar

**Affiliations:** Department of Cognitive and Computational Neuroscience, National Brain Research Center, Gurugram, Haryana 122051, India; Department of Psychiatry, Yale University School of Medicine, New Haven, CT 06519, USA; Monell Chemical Senses Center, Philadelphia, PA 19104, USA; National Drug Dependence Treatment Centre, All India Institute of Medical Sciences, New Delhi, Delhi 110029, India; National Drug Dependence Treatment Centre, All India Institute of Medical Sciences, New Delhi, Delhi 110029, India

**Keywords:** alcohol use disorder, machine learning, functional connectivity, olfactory processing, anxiety

## Abstract

Alcohol use disorder (AUD) often emerges in adolescence and young adulthood, yet early neural predictors remain scarce. The olfactory system, through its connections with limbic and reward circuits, may provide a novel window into compulsive alcohol use. These brain regions are altered in AUD, but the role of olfactory pathways is poorly understood. Anxiety, a common comorbidity in AUD, may further modulate these circuits. This study investigates whether anxiety moderates the relationship between olfactory–reward brain connectivity and risky alcohol use and dependence. We analysed multimodal data from 1003 participants (22–36 years) in the Human Connectome Project. Resting-state functional MRI was used to compute functional connectivity among 41 regions implicated in olfaction, emotion, memory and reward. Odour identification scores were used to isolate connectivity features most correlated with olfactory function. These features served as predictors in ElasticNet and LASSO classification models for three alcohol-related outcomes: (i) lifetime *Diagnostic and Statistical Manual of Mental Disorders (DSM)-IV* alcohol dependence, (ii) past-year risky drinking and (iii) past-week risky drinking. Models were trained on a Synthetic Minority Over-sampling Technique (SMOTE)-balanced subset (*n* = 704) and validated on a propensity score-matched holdout dataset (*n* = 83). *Post hoc* logistic regression examined anxiety (DSM scores) as a moderator of connectivity–outcome associations. Connectivity between the precuneus–cingulate–accumbens and lateral occipital–hippocampal regions predicted lifetime alcohol dependence (odds ratio = 1.12, *P* < 0.001). Models showed strong performance (area under the curve > 0.90 training; >0.80 validation). Anxiety moderated key connections: it attenuated the risk effect of memory–reward circuitry and amplified risk via inferior frontal–parietal connectivity. Odour identification scores did not differ across alcohol risk groups. Model performance replicated in a propensity-matched validation set. Our findings reveal a novel olfactory–limbic circuit predictive of alcohol dependence in young adults. Anxiety modulates these pathways, suggesting dynamic brain–behaviour interactions that may underlie individual vulnerability and resilience. This framework supports the integration of sensory, emotional and transdiagnostic features in neurobiological models of addiction.

## Teaser

Agarwal *et al*. report that brain connectivity within olfactory networks, together with anxiety, predicts vulnerability to alcohol dependence. Using functional MRI and behavioural data, they show that altered smell-related brain circuits, moderated by anxiety, can serve as potential markers for risky drinking and alcohol-related problems.

## Graphical Abstract

**Graphical Abstract:**
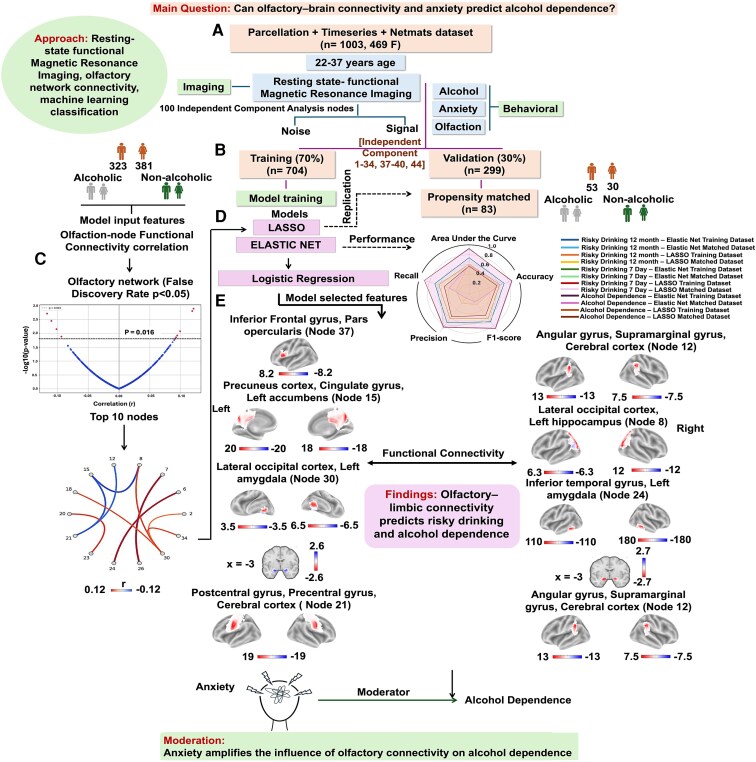

## Introduction

Alcohol use disorder (AUD) remains one of the most prevalent and persistent psychiatric conditions, with onset often emerging during adolescence and young adulthood, a critical developmental window for intervention.^1^ Alcohol use initiation, drinking propensity and AUD represent related yet distinct developmental processes.^2^ Alcohol use often begins in adolescence, driven by curiosity, social context or experimentation,^3^ whereas propensity to drink reflects stable behavioural tendencies such as sensation seeking, stress-driven drinking or affect regulation.^4,5^ In contrast, AUD is a chronic, relapsing condition marked by impaired control, craving and continued use despite harm. While early initiation increases the likelihood of later AUD,^5^ not all early users progress to problematic use,^6^ underscoring the need to differentiate these constructs when studying risk pathways. Recent advances in machine learning have shown promise in predicting AUD risk by integrating behavioural, cognitive and neurobiological data and their interactions. Predictive models incorporating multimodal features (such as personality traits, inhibitory control and reward-related brain activity) can explain up to 26% of variance in future alcohol use,^7^ with robust support between externalizing symptoms during adolescence and the emergence of AUD in early adulthood. Internalizing symptoms during adolescence, such as anxiety, share a more complex relationship with the emergence of AUD in adulthood.^8^ One hypothesis is that this complex relationship is related to differences in brain structure in emotional and memory areas, such as reduced anterior cingulate volume and enlarged nucleus accumbens, which have been identified as predictors of substance use initiation.^9^ Behavioural patterns, such as solitary drinking during adolescence, forecast AUD symptoms up to 17 years later, with a 35–60% increased risk compared to social drinkers.^10^ These findings underscore the urgency of identifying neurobehavioural predictors in young adults, a population especially vulnerable to entrenched drinking trajectories that can progress in AUD.^11^ Notably, gender-specific neural correlates of alcohol use risk have emerged: for example, increased connectivity in medial frontal, salience and sensorimotor networks predicts higher risk in female young adults, whereas inhibitory control-related networks are more predictive in males.^12^ These advances suggest that individual differences in brain connectivity can guide early, targeted prevention strategies, yet much of the variance in AUD vulnerability remains unexplained.

One underexplored yet promising area of variability is olfactory ability. Olfactory function shows a dynamic relationship with alcohol use, wherein odour cues may facilitate early drinking behaviours, but chronic alcohol exposure leads to diminished odour sensitivity and reduced olfactory influence. Moreover, intact olfactory systems facilitate the detection of alcohol-related cues, which strongly activate reward-related brain areas like the nucleus accumbens and ventral tegmental area promoting craving and reinforcing consumption patterns.^13,14^ In contrast, chronic alcohol use impairs odour discrimination, detection and identification, changes that may blunt environmental feedback and reinforce maladaptive drinking cycles.^15,16^ Despite this, olfaction is rarely considered in predictive models of AUD, even though its neural basis overlaps with emotional and reward networks.^17,18^ Clarifying the role of olfactory and emotion-related brain connectivity may improve our ability to predict AUD risk in young adults.

Critically, olfactory processing is modulated by anxiety, a psychiatric condition that frequently co-occurs with AUD. Anxiety states enhance olfactory sensitivity and alter brain connectivity between olfactory and emotion-processing regions, particularly the amygdala.^19,20^ Given that anxiety also predicts alcohol misuse^21^ and that alcohol use can exacerbate anxiety,^22^ this interaction may heighten reactivity to alcohol-associated olfactory cues and increase susceptibility to AUD.^23^ While our previous work has identified gustatory and olfactory networks as predictors of alcohol preference and consumption,^24^ the moderating role of anxiety on odour-related brain connectivity in relation to AUD risk remains unexamined. In this study, we address this gap by examining whether brain connectivity patterns in olfactory and emotion-related networks can predict alcohol dependence risk in young adults and whether anxiety symptoms influence this relationship. We leverage the Human Connectome Project (HCP) ‘PTN’ dataset, which has been widely used in prior work to relate individual differences in functional brain connectivity to various behavioural and cognitive variables such as education, tobacco use, intelligence, reading ability and personality traits^25,26^ (HCP MegaTrawl). We hypothesized that elevated anxiety symptoms would enhance the predictive strength of olfactory–limbic network connectivity for alcohol use risk, thereby identifying a neural mechanism by which anxiety may potentiate vulnerability to AUD.

## Materials and methods

### Dataset information

We used resting-state fMRI (rs-fMRI) data from the Human Connectome Project (HCP) Young Adult S1200 PTN dataset,^27^ which includes 1003 participants aged 22–37 (469 female). Imaging was performed on a 3T Siemens scanner using a multiband GE-EPI sequence [TR = 0.72 s, voxel size = 2 mm^3^, 1200 volumes per scan (15 min)]. Each participant completed four runs [two with right-to-left (RL), two with left-to-right (LR) phase encoding]. We used the PTN pipeline outputs (parcellation, timeseries and netmats) to ensure consistent, high-quality preprocessing for rs-fMRI data across participants. Further acquisition and preprocessing details are published elsewhere.^28,29^ All participants provided informed consent, and the study was approved by the Washington University Institutional Review Board (IRB# 201204036; Title: ‘Mapping the Human Connectome: Structure, Function and Heritability’).

### Functional connectivity matrices

For each participant, we analysed ICA-based functional connectivity matrices (100 × 100), derived from HCP dual-regression outputs (as in Smith *et al*.^27^, 2015). We focused only on components previously validated as signal using standard spatial and temporal quality control criteria.^24,30^ The current analyses use only the previously classified and network-labelled ICs as in Agarwal *et al*.^24^ (2025). The upper triangle of each matrix (excluding diagonal and redundant entries) was used as the feature space. Node identity was determined by overlapping ICA spatial maps with Harvard-Oxford anatomical atlases (thresholded at 25%; 2 mm MNI152 space) and identifying regions with peak voxel loadings per component.^24^ The process includes generation of binary masks for each anatomical region, extraction of voxel values from the ICA component map and computation of the mean ICA loading within the region:


$$\mathrm{Mean}\;\mathrm{ICA}\;\mathrm{loading}=1/N\sum_{j}N={\;}_{1}x_{i}$$

where *x_i_* is the ICA value at voxel *i* and *N* is the number of voxels in that region. Next, we identified regions with the maximum or near-maximum absolute mean ICA loading within each component. These high-loading regions were considered to most strongly represent the spatial pattern of the corresponding component and were used for anatomical labelling and interpretation in subsequent analyses.

### Behavioural measures

#### Odour identification

We used age-adjusted scores (Odor_AgeAdj) from the NIH Toolbox Odor Identification Test^31^ in the HCP sensory data. This nine-item scratch-and-sniff, forced-choice test, administered by a trained examiner, measures the ability to identify common odours (e.g. lemon, coffee and smoke from four picture–word options presented on a tablet). Total score is the sum of correctly identified odours and ranges from 0 to 9, with chance performance around 2. Lower scores indicate olfactory dysfunction. Scores were used as both predictors and to select functional connectivity features for modelling.

#### Alcohol outcomes

We derived three alcohol-related outcomes:

- **Lifetime alcohol dependence**: we used the variable SSAGA_Alc_D4_Dp_Dx from the Semi-Structured Assessment for the Genetics of Alcoholism (SSAGA) to identify individuals who met criteria for *DSM-IV* alcohol dependence at any point in their lifetime (1, no; 5, yes as coded in HCP dataset).
- **Past-year risky drinking**: we used the binary variable Risky_12Month to identify participants engaging in risky alcohol use in the past year based on either high average drink per drinking day (SSAGA_Alc_12_Drinks_Per_Day ≥ 4) or high drinking frequency (SSAGA_Alc_12_Frq ≥ 4), reflecting sustained heavy alcohol use.
- **Past-week risky drinking**: we used the binary variable Risky_Drinks_7days to identify participants as engaging in risky alcohol use if they reported consuming ≥14 drinks or ≥4 drinking days in the past week. These thresholds align with NIAAA guidelines for risky weekly consumption patterns (https://www.niaaa.nih.gov/health-professionals-communities/core-resource-on-alcohol/basics-defining-how-much-alcohol-too-much).

#### Anxiety symptoms

Anxiety symptom severity was assessed using the DSM-Oriented Anxiety Problems T-score (DSM_Anxi_T) calculated in this population using the Achenbach Adult Self-Report (ASR) instrument,^32^ retrieved from the HCP data. The DSM_Anxi_T score reflects age- and gender-adjusted symptom severity, allowing standardized comparisons across individuals. Higher scores indicate greater levels of anxiety-related problems based on self-report. The DSM_Anxi_T scores were used to investigate potential moderation effects of anxiety on the relationship between brain connectivity and alcohol-related behaviours.

#### Covariates

Participants’ age (years), gender (numerically coded 0, female, and 1, male) and Fagerström test for nicotine dependence (FTND; screener for nicotine dependence with six questions) scores were assessed as covariates in all analyses as these variables are linked to chemosensory ability and anxiety proneness.

### Statistical analysis for connectivity-feature selection

#### Brain connections correlated with odour identification scores

To identify olfactory-relevant brain connections, we correlated odour identification scores with each of the selected ICA–ICA connectivity features, using Pearson correlations adjusted for covariates (age, gender, FTND, anxiety). Significance was corrected using the Benjamini–Hochberg false discovery rate (BH-FDR) multiple comparison. The top 10 connections were selected based on the strength of association (FDR-adjusted *P*-values) for downstream modelling.

#### Behavioural analyses: odour and alcohol outcomes

We first assessed whether individual differences in olfactory identification scores were associated with alcohol-related outcomes and whether this relationship was moderated by anxiety. For binary alcohol variables (past-year and weekly risky drinking, alcohol dependence), group differences in odour scores were assessed using *t*-tests or Wilcoxon rank-sum tests, depending on normality (Shapiro–Wilk test). For continuous alcohol measures (e.g. number of drinks per week), we used Pearson correlations. The Benjamini–Hochberg false discovery rate (FDR) method was used to correct for multiple comparisons.

#### Partitioning and validation strategy

We split the full dataset into a 70% training set (*n* = 704) and 30% holdout validation set (*n* = 299) to build and evaluate predictive models. This approach minimizes overfitting and ensures that model performance is assessed on unseen data, providing a more accurate estimate of generalizability. A stratified sampling approach was used to preserve class proportions via the createDataPartition() function from the caret package (http://zevross.com/blog/2017/09/19/predictive-modeling-and-machine-learning-in-r-with-the-caret-package/). To assess model generalizability, we created a covariate-balanced validation set (*n* = 83) from our validation set (*n* = 299) using propensity score matching via the nearest-neighbour method from the MatchIt package (https://dlab.berkeley.edu/news/introduction-propensity-score-matching-matchit). Propensity scores were estimated using logistic regression based on key covariates: age, gender, odour identification score, anxiety symptoms and FTND scores. Participants with missing values on any of these matching variables were excluded prior to matching (*n* = 48), resulting in an effective sample of 251 participants eligible for matching. Of these, 83 were successfully matched into a balanced validation subset. This complete-case approach minimizes confounding and mimics a quasi-experimental design, allowing a fairer and more robust evaluation of model performance in a demographically and clinically comparable subgroup. We compared demographic and behavioural characteristics between these subsets (training and propensity matched) including age, gender, FTND score, odour identification scores and alcohol drinking behaviours (lifetime alcohol dependence, past-year risky drinking and past-week risky drinking) between the training and propensity-matched subsets using appropriate statistical tests (Welch’s *t*-test or Chi-squared test as applicable).

#### Model training and evaluation

We built predictive models for the three alcohol outcomes, with covariates using LASSO, ElasticNet and logistic regression, implemented via 5-fold cross-validation. Models were trained on the top 10 odour-linked connections plus covariates (age, gender, FTND, anxiety). To address class imbalance, we applied the Synthetic Minority Over-sampling Technique (SMOTE) within each fold (smotefamily package). Final models were trained on the full SMOTE-balanced training set and tested on the independent propensity-matched validation set. All modelling was conducted in R (v4.2.2) using caret, glmnet, e1071, pROC and broom. Model performance was evaluated using standard classification metrics: area under the receiver operating characteristic curve (AUC), accuracy, precision, recall, F1 score and specificity.^33^ AUC was the primary measure of discriminative ability, with values above 0.70 considered acceptable and above 0.80 considered strong. These metrics were calculated separately for both the training and validation datasets to assess generalizability and potential overfitting. A default probability threshold of 0.5 was used to assign binary class labels. We also evaluated class balance and model generalizability by reporting performance metrics across both datasets. Predictive features identified via ElasticNet regularization were further examined through *post hoc* logistic regression analyses to determine main effects and interaction with anxiety symptoms. Importantly, replication was assessed through predictive performance, rather than re-estimation of model coefficients. That is, the same trained model was applied to the validation set, and generalization was evaluated by how well it predicted outcomes in a propensity-matched sample. This strategy avoids instability caused by small validation sample sizes and emphasizes robust, generalizable learning.

#### Feature interpretation and moderation analysis

To interpret predictive features, we extracted non-zero coefficients from the LASSO and ElasticNet models. We then conducted *post hoc* logistic regressions on the SMOTE-balanced training set, adjusting for covariates (age, gender, FTND, anxiety), to estimate odds ratios (OR), 95% confidence intervals (CI) and *P*-values. To test moderation by anxiety, we created interaction terms between each connectivity feature and DSM_Anxi_T and included them in separate logistic models. These moderation analyses were restricted to the training set to avoid overfitting.

## Results

### Characteristics of training and validation dataset

No significant difference in demographic or clinical characteristics emerged between the participants in the training (*n* = 704) and propensity-matched validation (*n* = 83) datasets. Specifically, no differences were seen in age, FTND scores or odour identification scores between the two cohorts. However, a higher percentage of males was identified in our matched validation compared to the training dataset (*χ*^2^ = 8.9, *P* = 0.002). Although propensity score matching aimed to balance key covariates (age, odour score, anxiety and FTND), a higher proportion of males was retained in the matched validation set. This likely reflects the original dataset’s sex distribution and the fact that matching prioritized multivariate balance rather than strict sex equivalence. We also compared the groups across alcohol drinking variables, weekly risky drinking, past-year risky drinking and lifetime alcohol dependence, and no significant differences were identified in their drinking patterns. These results confirmed covariate and alcohol intake balance across the two partitioned groups (see Table 1).

**Table 1:** Participant characteristics in training and validation datasets

Participant characteristics	Training dataset	Propensity matched validation dataset	*t*-test or Chi-squared test
Age (in years)			
*N*	704	83	Effect size = −0.15; *P* = 0.21
Mean	28.5	29.1	
SD	3.6	3.8	
Anxiety			
*N*	703	297	Effect size = −0.10; *P* = 0.38
Mean	53.29	53.81	
SD	5.17	5.27	
Fagerström test for nicotine dependence (FTND) score			
*N*	174	84	Effect size = 0.23; *P* = 0.07
Mean	2.18	1.73	
SD	1.90	1.89	
Odour identification score			
*N*	704	299	Effect size = 0.19; *P* = 0.07
Mean	97.97	95.60	
SD	12.26	11.17	
Gender			
Male	323 (45.9%)	53 (63.9%)	*χ* ^2^ (df = 1) = 8.9, *P* = 0.002
Female	381 (54.1%)	30 (36.1%)	
Risking drinking for 7 days			
No	560 (81.4%)	61 (73.5%)	*χ* ^2^ (df = 1) = 2.46, *P* = 0.11
Yes	128 (18.6%)	22 (26.5%)	
Risky drinking for 12 months			
No	139 (20.8%)	16 (19.5%)	*χ* ^2^ (df = 1) = 0.02, *P* = 0.89
Yes	528 (79.2%)	66 (80.5%)	
Alcohol dependence			
No	661 (94%)	83 (100%)	*χ* ^2^ (df = 0) = NA
Yes	42 (6%)	0 (0%)	

### Association of odour identification scores with brain connections and alcohol drinking behaviour

Pearson correlation analyses between age-adjusted odour identification scores and resting-state functional connectivity revealed 30 significant brain connections. These associations spanned a range of functional systems, with correlation coefficients ranging from *r* = −0.12 to *r* = 0.12, suggesting a distributed network of regions linked to olfactory identification performance. The strongest positive association was observed between Node 26 and Node 7 (*r* = 0.12, *P* = 0.001), involving connectivity between the frontal operculum and paracingulate gyrus (Node 26) and the supramarginal gyrus (Node 7). These regions are implicated in interoceptive awareness, attention reorientation and multisensory integration.^34^ The strongest negative correlation emerged between Node 15 and Node 8 (*r* = −0.12, *P* = 0.001), representing a link between the precuneus cortex, cingulate gyrus and left nucleus accumbens (Node 15) and the lateral occipital cortex and left hippocampus (Node 8), highlighting the involvement of reward and memory systems in olfactory processing.^17,18^ Several other connections implicated olfactory, limbic and higher-order sensory regions: Node 30–Node 24 [*r* = 0.091, *P* = 0.015: lateral occipital cortex and left amygdala (Node 30) with the inferior temporal gyrus and left amygdala (Node 24)], suggesting visual–limbic integration; Node 25–Node 15 [*r* = 0.079, *P* = 0.037: connectivity between the occipital pole and right amygdala (Node 25) and precuneus cortex and accumbens (Node 15)], both key to emotion and reward circuits^35^; Node 21–Node 12 [*r* = −0.101, *P* = 0.007: linking the sensorimotor cortex (Node 21: precentral and post-central gyri) and temporoparietal regions (Node 12: angular and supramarginal gyri)]; Node 15–Node 2 [*r* = 0.084, *P* = 0.026: integrating the precuneus–cingulate–accumbens system (Node 15) with the angular and middle temporal gyri (Node 2)], both of which contribute to semantic memory and social cognition^36^; Node 44–Node 11 [*r* = 0.081, *P* = 0.032: linking the middle temporal and inferior frontal gyri with the left accumbens (Node 44) to the occipital fusiform and lingual gyri (Node 11)], suggesting a visual–emotional association^37^; Node 14–Node 11 [*r* = 0.087, *P* = 0.021: coupling between the inferior temporal gyrus and left caudate (Node 14) and the lingual and fusiform areas (Node 11)], both involved in visual object recognition and motivational salience. No significant differences in odour identification scores were observed between risky and non-risky drinkers (weekly or past-year alcohol consumption), as well as alcohol-dependent and non-dependent individuals (all *P* > 0.11). Correlation analyses between continuous alcohol use variables and odour identification scores also yielded non-significant associations (all *P* > 0.23). These results suggest that odour identification ability may not serve as a discriminative marker for alcohol use in young adults. This could indicate that olfactory impairment is either not present or not yet detectable at this stage. Overall, these findings imply that alcohol intake, whether examined categorically or dimensionally, does not exert a measurable impact on olfactory identification performance in this population.

### Model performance on SMOTE-balanced training set

**Figure 1:**
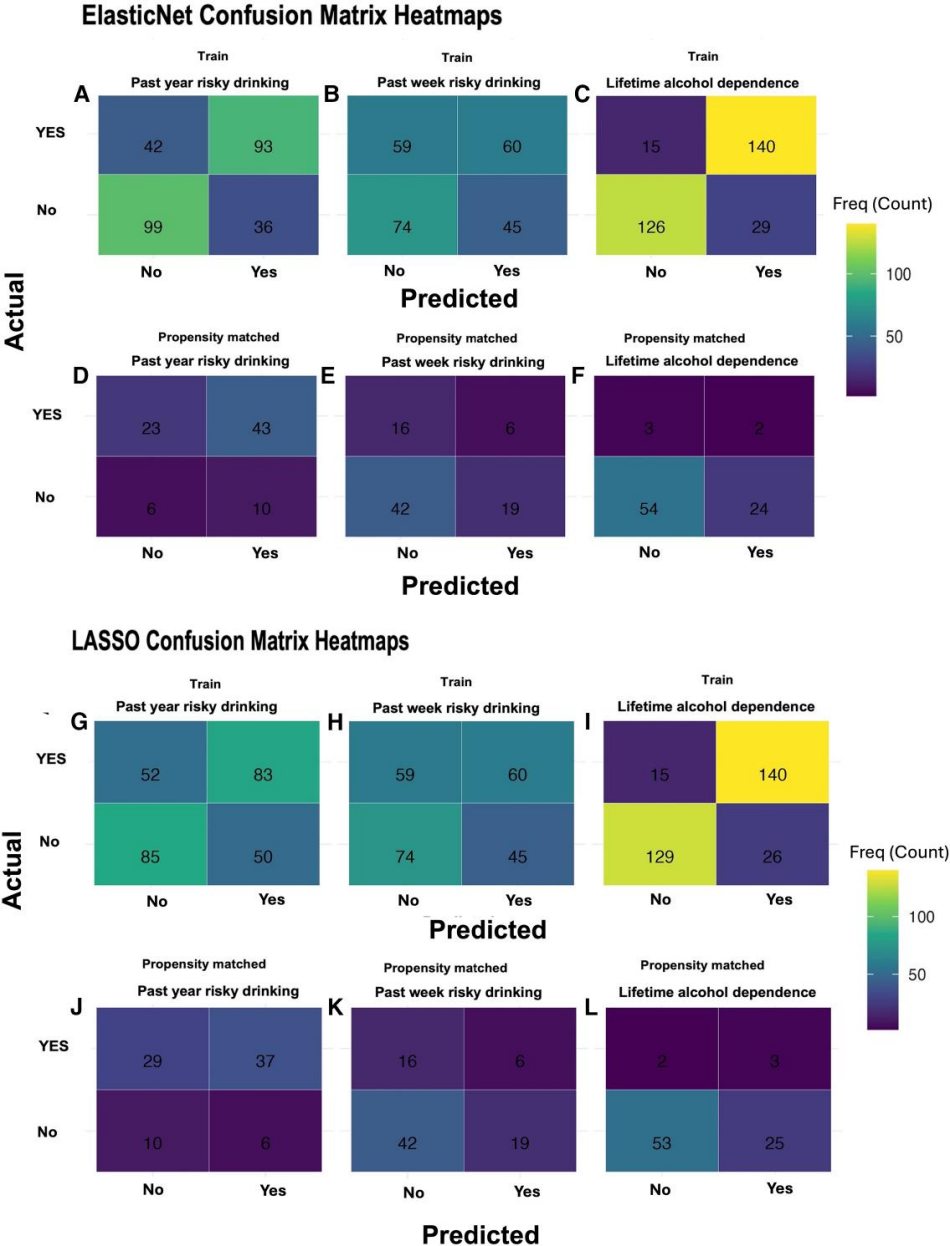
**Confusion matrix heatmaps for ElasticNet and LASSO models predicting alcohol-related outcomes.** Panels **A–F** show ElasticNet results, and Panels **G–L** show LASSO results. For each model, confusion matrices are presented for the SMOTE-balanced training dataset (*top rows*: **A–C**, **G–I**) and the propensity-matched validation dataset (*bottom rows*: **D–F**, **J–L**) across three binary alcohol-related outcomes: past-year risky drinking, past-week risky drinking and lifetime alcohol dependence. ElasticNet (**A–F**) training: (**A**) past-year risky drinking, (**B**) past-week risky drinking, (**C**) lifetime alcohol dependence. Validation: (**D**) past-year risky drinking, (**E**) past-week risky drinking, (**F**) lifetime alcohol dependence. LASSO (**G–L**) training: (**G**) past-year risky drinking, (**H**) past-week risky drinking, (**I**) lifetime alcohol dependence. Validation: (**J**) past-year risky drinking, (**K**) past-week risky drinking, (**L**) lifetime alcohol dependence. Each panel displays a confusion matrix with cell colours representing the frequency (count) of true positives, false positives, true negatives and false negatives. Lighter colours indicate higher frequencies. The experimental unit is the individual participant (training, *n* = 704; validation, *n* = 83). Values represent raw classification frequencies; no inferential tests were applied. The heatmap colour scale represents frequency (count).

Predictive models trained on the SMOTE-balanced training dataset exhibited high performance for lifetime alcohol dependence and past-year risky drinking (Fig. 1: Panels A–C for ElasticNet and G–I for LASSO). For lifetime alcohol dependence, LASSO achieved 86.8% accuracy, 90.3% recall and an AUC of 0.92 (Fig. 1I), while ElasticNet showed comparable performance (85.8% accuracy; 90.3% recall; AUC = 0.93; Fig. 1C). For past-year risky drinking, ElasticNet outperformed LASSO (ElasticNet, 71.1% accuracy and 68.9% recall, AUC = 0.74; LASSO, 62.2% accuracy, 61.5% recall, AUC = 0.61; Fig. 1A versus 1G). In contrast, past-week risky drinking remained challenging for both methods, with modest predictive performance (accuracy ≈ 57%, AUC ≈ 0.57; Fig. 1B and H). Propensity-matched validation results are shown in Fig. 1 Panels D–F for ElasticNet and J–L for LASSO, demonstrating reduced but still meaningful discrimination for past-year risky drinking and lifetime alcohol dependence, consistent with the more conservative matched sample (see Table 2).

**Table 2:** Comparison of model performance metrics for ElasticNet and LASSO across training and propensity-matched validation sets

	ElasticNet		LASSO	
	Train	Propensity matched	Train	Propensity matched
Risky drinking past 12 months				
AUC	0.74	0.55	0.61	0.61
Accuracy	0.71	0.6	0.62	0.57
F1 score	0.7	0.72	0.62	0.68
Precision	0.72	0.81	0.62	0.86
Recall	0.69	0.65	0.61	0.56
Risky drinking 7 days				
AUC	0.57	0.6	0.57	0.6
Accuracy	0.56	0.58	0.56	0.58
F1 score	0.54	0.26	0.54	0.26
Precision	0.57	0.24	0.57	0.24
Recall	0.5	0.27	0.5	0.27
Alcohol dependence				
AUC	0.93	0.58	0.92	0.92
Accuracy	0.86	0.67	0.87	0.87
F1 score	0.86	0.2	0.87	0.87
Precision	0.83	0.1	0.84	0.84
Recall	0.9	0.4	0.9	0.9

### Feature interpretation and *post hoc* logistic regression analysis (training set only)

*Post hoc* binomial logistic regression analyses on the SMOTE-balanced training dataset were conducted across all three alcohol intake outcomes. The top 10 connections associated with odour identification scores were used as predictors in this model. These analyses aimed to estimate OR, *P*-values and CI for selected predictors while adjusting for relevant covariates. Importantly, *post hoc* regression was not done on the validation set due to small sample size and associated risks of unstable parameter estimates. Lifetime alcohol dependence: ElasticNet *post hoc* logistic regression identified two statistically significant predictors—the connectivity between the precuneus–cingulate–accumbens cluster and the lateral occipital cortex–hippocampus (Node 15–Node 8), which increased the odds of dependence (OR = 1.12, *P* < 0.001), and an interaction between inferior frontal gyrus (pars opercularis) and angular/supramarginal gyrus connectivity (Node 37–Node 12) with anxiety scores, which also contributed positively to risk (OR = 1.01, *P* = 0.01). The LASSO model corroborated the predictive value of Node 15–Node 8 (OR = 1.01, *P* < 0.001) and additionally identified two protective factors: connectivity between the lateral occipital cortex–amygdala and inferior temporal gyrus–amygdala (Node 30–Node 24) (OR = 0.001, *P* < 0.001), post-central gyrus–supramarginal gyrus (Node 21–Node 12) and paracingulate gyrus–supramarginal gyrus (Node 26–Node 7) was associated with reduced dependence risk (Supplemental Table S1). Past-year risky drinking: ElasticNet models revealed significant negative associations with connectivity between the frontal operculum–paracingulate gyrus and supramarginal gyrus (Node 26–Node 7; OR = 0.43, *P* = 0.003) and between the inferior temporal gyrus–caudate and occipital fusiform gyrus–lingual gyrus (Node 14–Node 11; OR = 0.93, *P* = 0.01). DSM anxiety also emerged as a strong negative predictor (OR = 0.61, *P* = 0.001), suggesting individuals with higher anxieties were less likely to engage in past-year risky drinking, potentially reflecting avoidant tendencies. In contrast, the LASSO model highlighted a significant interaction between Node 15–Node 8 and anxiety (OR = 0.99, *P* = 0.004), implying that increased anxiety moderated the risk-enhancing effect of this connectivity feature. This points to a nuanced, context-dependent role of memory–reward circuitry in sustained drinking behaviour (see Supplemental Table S2). Weekly risky drinking: for recent drinking behaviour, neither ElasticNet nor LASSO models produced statistically significant predictors in *post hoc* regressions. This result is consistent with prior findings suggesting higher variability and behavioural instability in short-term alcohol use patterns, reducing the reliability of neural predictors for this outcome.

### Model replication via propensity-matched validation set

To assess model generalizability, trained classifiers were evaluated on an independent propensity-matched validation set (*n* = 83). No *post hoc* logistic regression was conducted on this set due to the small sample size and high variance in coefficient estimates. Instead, replication was assessed through predictive performance metrics (accuracy, AUC, F1 score).

**Figure 2:**
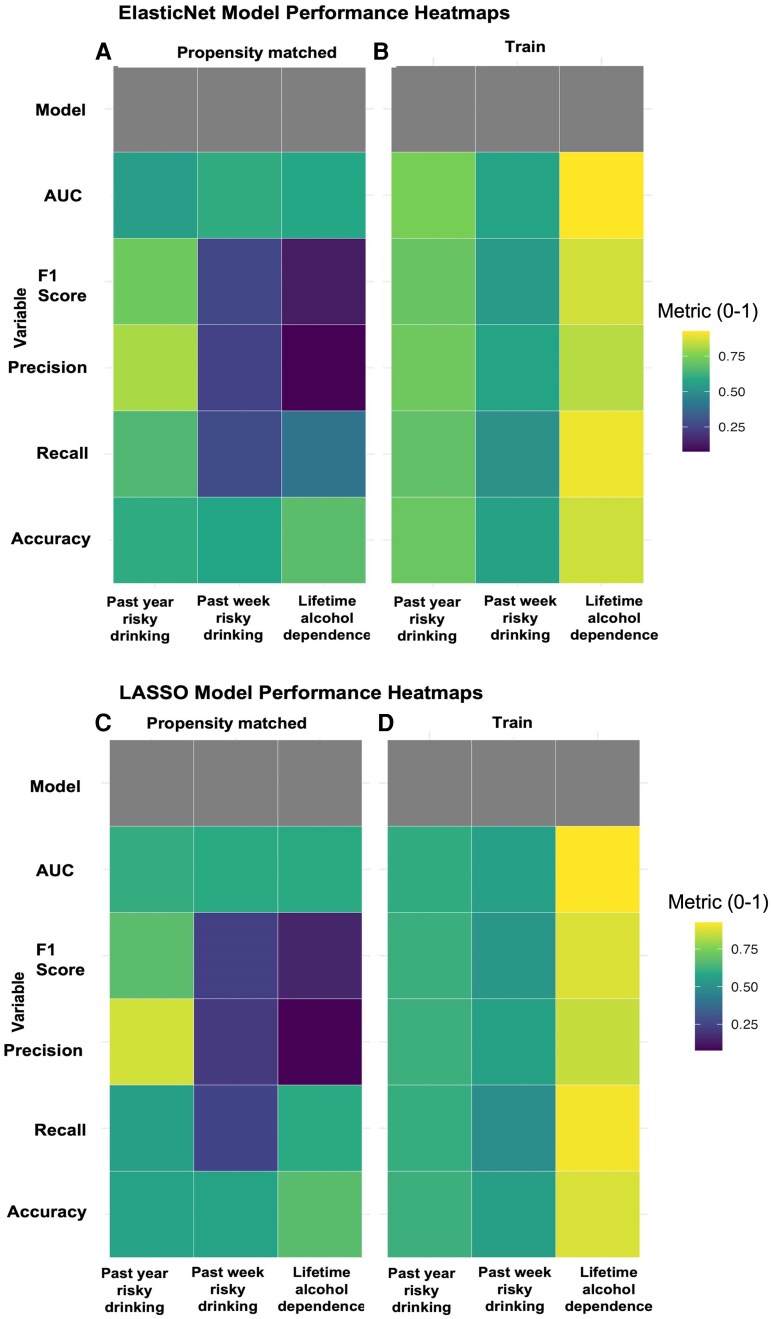
**Model performance heatmaps for ElasticNet and LASSO models predicting alcohol-related outcomes.** Panels **A–D** present model-level performance metrics for three binary alcohol-related outcomes: past-year risky drinking, past-week risky drinking and lifetime alcohol dependence evaluated in the propensity-matched validation dataset and the SMOTE-balanced training dataset. ElasticNet (**A–B**): **A** shows validation-set performance, and **B** shows training-set performance across all outcomes. LASSO (**C–D**): **C** shows validation-set performance, and **D** shows training-set performance across all outcomes. Each heatmap summarizes five performance metrics: area under the curve (AUC), F1 score, precision, recall and accuracy. Cell colours reflect the metric value (0–1), with lighter colours indicating higher performance. Training-set values represent cross-validated estimates from repeated 10-fold cross-validation, whereas validation results reflect held-out evaluation in the covariate-matched sample. The experimental unit is the individual participant (training, *n* = 704; validation, *n* = 83). These heatmaps provide a high-level summary of model performance across outcomes and datasets. No inferential statistical tests were applied; values represent model-derived performance metrics. The heatmap colour scale represents the metric value (0–1).

In the propensity-matched validation sample, both ElasticNet and LASSO showed the strongest performance for lifetime alcohol dependence, with comparable overall accuracy and discrimination (Fig. 2A and C). ElasticNet demonstrated slightly higher AUC, whereas LASSO showed marginally better recall, indicating similar but complementary performance profiles across the two models. For past-year risky drinking, ElasticNet again showed more balanced performance, with higher recall and F1 score, while LASSO exhibited higher precision but reduced sensitivity (Fig. 2A and C). This pattern is consistent with greater stability of ElasticNet in identifying risky-drinking cases in the matched sample. Prediction of past-week risky drinking remained modest for both models, with lower F1 scores and accuracy relative to the other outcomes (Fig. 2A–D), consistent with the training set results. Overall, the performance heatmaps and model summaries presented in Fig. 2 and Table 2 demonstrate meaningful replication of training-set findings in an independent, covariate-matched validation sample, particularly for lifetime alcohol dependence and past-year risky drinking.

## Discussion

We set out to examine whether intrinsic brain connectivity in olfactory and emotion-related networks could identify individuals at elevated risk for alcohol dependence. Strikingly, we found that anxiety symptoms modulate this association: specific brain circuits linking sensory and reward systems were predictive of alcohol misuse, but the strength and direction of these associations varied systematically with anxiety levels. This points to a novel neurobehavioural pathway, rooted in how the brain processes odour-related information, that may underlie vulnerability to alcohol dependence in young adults.

Our data-driven modelling revealed that connectivity patterns involving the amygdala, nucleus accumbens, hippocampus and associated temporal and frontal cortices were most strongly associated with odour identification, as previously reported in the literature in smaller samples.^38^ These networks, implicated in emotional salience, reward valuation and memory processing, have also been implicated in addiction circuits.^39,40^ Although our analyses focus on olfactory–limbic connectivity, it is important to acknowledge that regions traditionally classified as ‘olfactory’ particularly the olfactory bulb and olfactory tubercle play well-established roles in reward processing, reinforcement learning and motivated behaviour. Animal studies show that the olfactory tubercle receives dopaminergic input from the ventral tegmental area and contributes to conditioned reinforcement, reward valuation and approach behaviour independent of pure sensory olfactory function.^41^ Therefore, functional alterations in these circuits in humans may reflect not only changes in odour perception but also broader motivational or reward-driven mechanisms that intersect with addiction vulnerability. This dual sensory-reward role suggests that the connectivity patterns identified here may index a more general mechanism of reinforcement sensitivity, attentional salience or affective memory, rather than olfactory processing alone. Future work incorporating multimodal olfactory tests, reward-related tasks and experimental manipulation of reinforcement contingencies will be necessary to distinguish sensory-specific versus reward-driven contributions to these neural signatures.

Yet, despite this robust neural engagement, odour identification scores did not differ significantly between individuals with and without alcohol dependence or risky drinking. The scratch-and-sniff identification task primarily measures semantic odour labelling and may not be sensitive enough to detect early or subtle olfactory deficits. More sensitive domains such as odour threshold or discrimination often reveal impairment earlier during alcohol misuse. Therefore, the absence of group differences does not necessarily indicate intact olfactory function in young adults; rather, impairment may be mild, heterogeneous or not detectable with the present method. Longitudinal studies and multimodal olfactory testing are needed to determine whether olfactory deficits emerge with prolonged or escalating alcohol use. While our modelling indicates that odour identification is subserved by neural circuits central to emotion, reward and memory networks also implicated in addiction, this does not necessarily imply that alcohol dependence or risky drinking leads to measurable deficits in odour identification. One interpretation is that functional connectivity may serve as a more sensitive indicator of vulnerability than behavioural performance alone, specifically odour identification. That is, the brain may encode risk well before it manifests behaviourally, a hypothesis supported by the strong predictive power of our models and revealed in other contexts.^42,43^ This suggests a dissociation between latent neural vulnerability and overt olfactory identification impairment. Testing other olfactory functions, such as odour sensitivity or intensity, could prove to be more informative in young samples as compared to older ones.^44,45^ We advance that this decoupling could serve as a window for early intervention, before behavioural signs emerge, and that including multiple olfactory functions perhaps via screening tests such as SCENTinel^46^ could be beneficial in these large-scale studies. Furthermore, while prior studies have focused on cue-reactivity to alcohol-related odours,^13,47^ our results demonstrate that resting-state olfactory–limbic connectivity alone, independent of active alcohol cues, is informative about long-term risk. This extends the conceptual framework of addiction vulnerability beyond exogenously triggered craving responses and into the intrinsic, trait-like architecture of sensory–emotional networks. Rather than reflecting momentary state shifts in reactivity to alcohol cues, these connectivity patterns may represent stable neural predispositions, pre-existing configurations that shape how sensory and affective information is processed, even at rest.

Notably, the most predictive features involved connections such as the precuneus cingulate–accumbens to lateral occipital–hippocampus (Node 15–Node 8). This circuit links self-referential and default mode regions with those involved in affective memory and reward sensitivity^48^ precisely the kinds of circuits hypothesized to contribute to maladaptive reinforcement learning in addiction.^49^ The fact that these connections emerged from an odour-based feature selection pipeline strengthens the case for multisensory integration, particularly involving olfaction, as a critical yet underrecognized dimension of addiction neuroscience. These results highlight the utility of brain connectivity features as predictive markers of early-stage alcohol misuse risk, preceding observable behavioural changes. Our LASSO and ElasticNet run on the SMOTE-balanced dataset revealed high classification accuracy, with AUCs above 0.90 for lifetime dependence. These findings support the hypothesis that functional connectivity patterns particularly in networks related to emotional memory and reward encode vulnerability for alcohol dependence. This aligns with findings from longitudinal studies of individuals with AUD, which have shown increased functional connectivity between negative affect and reward/incentive salience networks, and a reduced synchronicity between executive control and reward systems.^50^ Together, these connectivity patterns reveal the greater influence of emotion and reward signals while lesser influence of self-control neural mechanisms on continued chronic alcohol use despite known negative consequences. Further in our *post hoc* logistic regression analyses, specific brain connections particularly between the precuneus–cingulate–accumbens and lateral occipital–hippocampus regions were observed as strong predictors of alcohol dependence. These positive associations highlight the important role played by self-referential, reward and memory systems in addiction vulnerability.^49^ Further there are some contrasting observations wherein protective associations were observed for connections involving the amygdala and inferior temporal regions, suggesting a potential regulatory function that may reduce dependence risk.

Our findings also highlight the regulatory role of anxiety in this framework. While anxiety has long been recognized as a comorbidity in AUD,^22^ its mechanistic role has remained unclear. We observed that in some cases, anxiety attenuated the association between odour-related brain connectivity and alcohol dependence, most notably within the Node 15–Node 8 memory–reward circuit. This counterintuitive pattern prompts several possibilities. One interpretation is that anxiety, under certain conditions, may function as a protective factor, potentially by enhancing interoceptive vigilance or amplifying risk aversion, thereby reducing impulsive or compulsive engagement with alcohol. Alternatively, this attenuation effect could be methodological an artefact of the anxiety scale’s properties, such as a restricted range, low variance or ceiling effects in the sample. Further work using dimensional, state-dependent or multimodal anxiety measures may help disentangle these possibilities. This effect may be context dependent. Prior work suggests that anxiety can heighten sensitivity to threat and increase avoidance behaviour,^51^ potentially interfering with impulsive or reward-driven actions like alcohol use. Our moderation results align with this interpretation. However, we also identified cases where anxiety amplified the relationship between brain connectivity and alcohol-related risk, such as in the interaction between the precuneus–cingulate–accumbens cluster and the lateral occipital cortex–hippocampus (Node 15–Node 8), as well as between the inferior frontal gyrus (pars opercularis) and angular/supramarginal gyrus (Node 37–Node 12). These bidirectional effects suggest that anxiety does not act as a uniform risk factor but rather as a dynamic moderator of brain–behaviour relationships, possibly exerting protective or exacerbating influences depending on the neural context. This complexity highlights the need to move beyond categorical views of anxiety and consider individual differences or clinical subgroups that may be particularly susceptible or resilient to alcohol-related risk in the presence of anxiety.

Our models performed robustly across both training and propensity-matched validation datasets, especially for lifetime alcohol dependence and past-year risky drinking (see Fig. 2). This generalizability, despite the small validation sample, underscores the stability of the identified predictive patterns. However, prediction for short-term (past week) risky drinking was consistently poor. This reinforces the hypothesis that stable neural predictors are better suited for modelling chronic or sustained alcohol use, not transient behaviour. Although the dataset does not include prospective longitudinal follow-up, our analyses were not limited to predicting the presence of an established AUD diagnosis. In addition to lifetime alcohol dependence, we modelled past-year risky drinking and past-week risky drinking, both of which capture current consumption patterns that are clinically recognized indicators of elevated future AUD risk. These measures reflect ongoing hazardous use rather than established dependence and are widely used as validated proxies of AUD vulnerability, particularly in young adult populations where risky drinking often precedes diagnostic conversion. Thus, our models identify neural and anxiety-related predictors not only of diagnosed dependence but also of subthreshold, high-risk drinking trajectories. Importantly, the strongest and most reliable connectivity predictors emerged for past-year risky drinking, supporting the view that the identified features reflect a neurobehavioural vulnerability profile rather than consequences of chronic dependence alone. While true longitudinal prediction of future AUD onset is beyond the scope of the HCP dataset, the inclusion of these graded risk phenotypes allows us to meaningfully infer markers associated with progressing towards AUD.

Several questions remain. First, to what extent are these odour-related connectivity features genetically or environmentally determined? Prior work from the HCP cohort suggests high heritability in intrinsic connectivity patterns,^52^ but the role of early life exposure, stress and learning history in shaping these networks is still unknown.^53^ Second, are these brain signatures specific to alcohol dependence, or do they generalize to other substance use or affective disorders involving similar circuitry? Future transdiagnostic studies may clarify specificity. Finally, can these findings be translated into clinically actionable tools? The successful replication in a covariate-matched independent sample underscores the potential for these models to be applied in broader clinical or research contexts, while also highlighting the importance of outcome-specific variability in prediction. Our results offer a conceptual foundation for integrating olfactory neuroimaging with behavioural and psychiatric assessments in early-risk screening, particularly in individuals with co-occurring anxiety. However, as the current study is cross-sectional and drawn from a relatively healthy, young and educated sample, broader clinical validation is needed.

Our study is not without limitations. The study is limited by the absence of alcohol-related odour panels in the HCP dataset. Since only common environmental odours (e.g. lemon, popcorn and natural gas) were provided, we were unable to evaluate whether individuals with alcohol use exhibit selective impairment or heightened sensitivity to alcohol-associated smells. This restricts the specificity of our conclusions. The cross-sectional design precludes causal inference: we cannot determine whether odour-related connectivity predisposes individuals to drink or reflects the neural consequence of prior use. Additionally, our sample lacks diversity in olfactory performance and severe AUD phenotypes, potentially constraining generalizability. Effect sizes, while statistically robust, remain modest suggesting that these features, although meaningful, explain only part of the variance in alcohol vulnerability. Additionally, issues of recall and classification biases may be operational in the diagnosis of alcohol dependence and risky alcohol use in this population. Future work should incorporate longitudinal designs, richer sensory testing (e.g. odour threshold and discrimination) and larger, clinically enriched samples. It will also be critical to examine whether interventions that reduce anxiety (whether pharmacological, behavioural or neuromodulatory) alter olfactory–limbic connectivity patterns and reduce alcohol use trajectories. If so, these brain–behaviour–emotion pathways could serve not just as biomarkers but as modifiable targets.

## Conclusion

In summary, our results indicate that odour identification ability may not serve as a discriminative marker for alcohol use in young adults, suggesting that olfactory impairment might either be absent or not yet detectable at this developmental stage. Odour-related brain connectivity (particularly within emotion, memory and reward networks) can predict vulnerability to alcohol dependence, and anxiety plays a context-dependent moderating role. This work uncovers a previously underappreciated sensory–emotional pathway in addiction neuroscience, laying the groundwork for novel predictive and preventive strategies, integrating sensory and psychological traits.

## Supplementary Material

fcag096_Supplementary_Data

## Data Availability

All analysis code used in this study is provided in Supplementary material. The data supporting the findings of this study are available from the corresponding author upon reasonable request, subject to institutional and ethical approvals. For any further information, please contact khushbu.agarwal@nbrc.ac.in

## References

[1] Brown AS, McGue M, Maggs J, et al A developmental perspective on alcohol and youths 16 to 20 years of age. Pediatrics. 2008;121(Supplement_4):S290–S310. 18381495 10.1542/peds.2007-2243D PMC2765460

[2] Chassin L, Sher JK, Hussong A, Curran P. The developmental psychopathology of alcohol use and alcohol disorders: Research achievements and future directions. Dev Psychopathol. 2013;25(4pt2):1567–1584. 24342856 10.1017/S0954579413000771 PMC4080810

[3] Petit G, Kornreich C, Verbanck P, Cimochowska A, Campanella S. Why is adolescence a key period of alcohol initiation and who is prone to develop long-term problem use?: A review of current available data. Socioaffect Neurosci Psychol. 2013;3(1):21890. 24693359 10.3402/snp.v3i0.21890 PMC3960066

[4] Doumas MD, Miller R, Esp S. Impulsive sensation seeking, binge drinking, and alcohol-related consequences: Do protective behavioral strategies help high risk adolescents? Addict Behav. 2017;64:6–12. 27533076 10.1016/j.addbeh.2016.08.003 PMC10662253

[5] Richmond-Rakerd SL, Slutske SW, Lynskey TM, et al Age at first use and later substance use disorder: Shared genetic and environmental pathways for nicotine, alcohol, and cannabis. J Abnorm Psychol. 2016;125(7):946–959. 27537477 10.1037/abn0000191 PMC5061603

[6] Ystrom E, Kendler SK, Reichborn-Kjennerud T. Early age of alcohol initiation is not the cause of alcohol use disorders in adulthood, but is a major indicator of genetic risk. A population-based twin study. Addiction. 2014;109(11):1824–1832. 24845951 10.1111/add.12620 PMC4192000

[7] Nees F, Tzschoppe J, Patrick JC, et al Determinants of early alcohol use in healthy adolescents: The differential contribution of neuroimaging and psychological factors. Neuropsychopharmacology. 2012;37(4):986–995. 22113088 10.1038/npp.2011.282 PMC3280646

[8] Dyer LM, Easey EK, Heron J, Hickman M, Munafò RM. Associations of child and adolescent anxiety with later alcohol use and disorders: A systematic review and meta-analysis of prospective cohort studies. Addiction. 2019;114(6):968–982. 30891835 10.1111/add.14575 PMC6563455

[9] Boer DO, Marroun EH, Franken HAI. Brain morphology predictors of alcohol, tobacco, and cannabis use in adolescence: A systematic review. Brain Res. 2022;1795:148020. 35853511 10.1016/j.brainres.2022.148020

[10] Creswell GK, Terry-Mcelrath MY, Patrick EM. Solitary alcohol use in adolescence predicts alcohol problems in adulthood: A 17-year longitudinal study in a large national sample of US high school students. Drug Alcohol Depend. 2022;238:109552. 35835632 10.1016/j.drugalcdep.2022.109552 PMC9639649

[11] Hingson WR, Zha W. Age of drinking onset, alcohol use disorders, frequent heavy drinking, and unintentionally injuring oneself and others after drinking. Pediatrics. 2009;123(6):1477–1484. 19482757 10.1542/peds.2008-2176

[12] Tschorn M, Lorenz CR, O’Reilly FP, et al Differential predictors for alcohol use in adolescents as a function of familial risk. Transl Psychiatry. 2021;11(1):157. 33664233 10.1038/s41398-021-01260-7 PMC7933140

[13] Kareken AD, Claus DE, Sabri M, et al Alcohol-related olfactory cues activate the nucleus accumbens and ventral tegmental area in high-risk drinkers: Preliminary findings. Alcohol Clin Exp Res. 2004;28(4):550–557. 15100605 10.1097/01.alc.0000122764.60626.af

[14] Oberlin GB, Dzemidzic M, Tran MS, et al Beer flavor provokes striatal dopamine release in male drinkers: Mediation by family history of alcoholism. Neuropsychopharmacology. 2013;38(9):1617–1624. 23588036 10.1038/npp.2013.91 PMC3717546

[15] Rupp IC, Fleischhacker WW, Hausmann A, Mair D, Hinterhuber H, Kurz M. Olfactory functioning in patients with alcohol dependence: Impairments in odor judgements. Alcohol and Alcoholism. 2004;39(6):514–519. 15456691 10.1093/alcalc/agh100

[16] Maurage P, Callot C, Philippot P, Rombaux P, Timary DP. Chemosensory event-related potentials in alcoholism: A specific impairment for olfactory function. Biol Psychol. 2011;88(1):28–36. 21718751 10.1016/j.biopsycho.2011.06.004

[17] Billot P-E, Andrieu P, Biondi A, Vieillard S, Moulin T, Millot J-L. Cerebral bases of emotion regulation toward odours: A first approach. Behav Brain Res. 2017;317:37–45. 27633559 10.1016/j.bbr.2016.09.027

[18] Gaeta G, Sullivan RM, Wilson DA Neural Mechanisms for Odor-Guided Behavior. Oxford University Press; 2023.

[19] Takahashi T, Itoh H, Nishikawa Y, et al Possible relation between olfaction and anxiety in healthy subjects. Psychiatry Clin Neurosci. 2015;69(7):431–438. 25605415 10.1111/pcn.12277

[20] Krusemark AE, Novak RL, Gitelman RD, Li W. When the sense of smell meets emotion: Anxiety-state-dependent olfactory processing and neural circuitry adaptation. J Neurosci. 2013;33(39):15324–15332. 24068799 10.1523/JNEUROSCI.1835-13.2013 PMC3782615

[21] Rosenström HT, Torvik AF. Social anxiety disorder is a risk factor for alcohol use problems in the National Comorbidity Surveys. Drug Alcohol Depend. 2023;249:109945. 37302357 10.1016/j.drugalcdep.2023.109945

[22] Smith JPR, C L. Anxiety and alcohol use disorders: Comorbidity and treatment considerations. Alcohol Res. 2012;34:414–431. 23584108 10.35946/arcr.v34.4.06 PMC3860396

[23] Anker J . Co-occurring alcohol use disorder and anxiety: Bridging psychiatric, psychological, and neurobiological perspectives. Alcohol Res. 2019;40(1):arcr.v40.1.03. 31886106 10.35946/arcr.v40.1.03 PMC6927748

[24] Agarwal K, Chaudhary S, Tomasi D, Volkow DN, Joseph VP. Prediction of alcohol intake patterns with olfactory and gustatory brain connectivity networks. Neuropsychopharmacology. 2025;50(7):1167–1175. 39962224 10.1038/s41386-025-02058-7 PMC12089591

[25] Liu W, Kohn N, Fernández G. Intersubject similarity of personality is associated with intersubject similarity of brain connectivity patterns. NeuroImage. 2019;186:56–69. 30389630 10.1016/j.neuroimage.2018.10.062

[26] Smith MS, Nichols ET, Vidaurre D, et al A positive-negative mode of population covariation links brain connectivity, demographics and behavior. Nat Neurosci. 2015;18(11):1565–1567. 26414616 10.1038/nn.4125 PMC4625579

[27] Smith MS, Beckmann FC, Andersson J, et al Resting-state fMRI in the Human Connectome Project. NeuroImage. 2013;80:144–168. 23702415 10.1016/j.neuroimage.2013.05.039 PMC3720828

[28] Essen VCD, Smith MS, Barch MD, Behrens EJT, Yacoub E, Ugurbil K. The WU-Minn Human Connectome Project: An overview. NeuroImage. 2013;80:62–79. 23684880 10.1016/j.neuroimage.2013.05.041 PMC3724347

[29] Barch MD, Burgess CG, Harms PM, et al Function in the human connectome: Task-fMRI and individual differences in behavior. NeuroImage. 2013;80:169–189. 23684877 10.1016/j.neuroimage.2013.05.033 PMC4011498

[30] Bhaganagarapu K, Jackson DG, Abbott FD. An automated method for identifying artifact in independent component analysis of resting-state fMRI. Front Hum Neurosci. 2013;7:343. 23847511 10.3389/fnhum.2013.00343 PMC3706880

[31] Dalton P, Doty LR, Murphy C, et al Olfactory assessment using the NIH Toolbox. Neurology. 2013;80(11_supplement_3):S32–S36. 23479541 10.1212/WNL.0b013e3182872eb4 PMC3662337

[32] Achenbach TM, Ivanova MY, Rescorla LA. Empirically based assessment and taxonomy of psychopathology for ages 1½-90+ years: developmental, multi-informant, and multicultural findings. Compr Psychiatry. 2017;79:4–18. 28356192 10.1016/j.comppsych.2017.03.006

[33] Powers D . Evaluation: from precision, recall and F-measure to ROC, informedness, markedness and correlation. *J Mach Learn Tech*. 2011;2:37263.

[34] Herman MA, Palmer C, Azevedo TR, Tsakiris M. Neural divergence and convergence for attention to and detection of interoceptive and somatosensory stimuli. Cortex. 2021;135:186–206. 33385747 10.1016/j.cortex.2020.11.019

[35] Cardinal NR, Parkinson AJ, Hall J, Everitt JB. Emotion and motivation: The role of the amygdala, ventral striatum, and prefrontal cortex. Neurosci Biobehav Rev. 2002;26(3):321–352. 12034134 10.1016/s0149-7634(02)00007-6

[36] Tanguay FA, Palombo JD, Love B, Glikstein R, Davidson SP, Renoult L. The shared and unique neural correlates of personal semantic, general semantic, and episodic memory. eLife. 2023;12:e83645. 37987578 10.7554/eLife.83645 PMC10662951

[37] Geday J, Gjedde A, Boldsen A-S, Kupers R. Emotional valence modulates activity in the posterior fusiform gyrus and inferior medial prefrontal cortex in social perception. NeuroImage. 2003;18(3):675–684. 12667845 10.1016/s1053-8119(02)00038-1

[38] Bestgen A-K, Schulze P, Kuchinke L. Odor emotional quality predicts odor identification. Chem Senses. 2015;40(7):517–523. 26142420 10.1093/chemse/bjv037

[39] Volkow DN, Michaelides M, Baler R. The neuroscience of drug reward and addiction. Physiol Rev. 2019;99(4):2115–2140. 31507244 10.1152/physrev.00014.2018 PMC6890985

[40] Koob FG, Volkow DN. Neurobiology of addiction: A neurocircuitry analysis. Lancet Psychiatry. 2016;3(8):760–773. 27475769 10.1016/S2215-0366(16)00104-8 PMC6135092

[41] Ikemoto S . Brain reward circuitry beyond the mesolimbic dopamine system: A neurobiological theory. Neurosci Biobehav Rev. 2010;35(2):129–150. 20149820 10.1016/j.neubiorev.2010.02.001 PMC2894302

[42] Lauharatanahirun N, Maciejewski FD, Kim-Spoon J, King-Casas B. Risk-related brain activation is linked to longitudinal changes in adolescent health risk behaviors. Dev Cogn Neurosci. 2023;63:101291. 37672817 10.1016/j.dcn.2023.101291 PMC10485595

[43] Dimakou A, Pezzulo G, Zangrossi A, Corbetta M. The predictive nature of spontaneous brain activity across scales and species. Neuron. 2025;113(9):1310–1332. 40101720 10.1016/j.neuron.2025.02.009

[44] Xu L, Liu J, Wroblewski EK, McClintock KM, Pinto MJ. Odor sensitivity versus odor identification in older US adults: Associations with cognition, age, gender, and race. Chem Senses. 2020;45(4):321–330. 32406505 10.1093/chemse/bjaa018 PMC7320224

[45] Sabiniewicz A, Krause M, Hummel T. Age-related decrease of odorant sensitivity for a selection of nine diverse molecules. European Archives of Oto-Rhino-Laryngology. 2025;282(5):2663–2668. 39994033 10.1007/s00405-025-09254-7 PMC12055947

[46] Parma V, Hannum EM, O’Leary M, et al SCENTinel 1.0: Development of a rapid test to screen for smell loss. Chem Senses. 2021;46:bjab012. 33773496 10.1093/chemse/bjab012 PMC8083606

[47] Stormark MK, Laberg CJ, Bjerland T, Nordby H, Hugdahl K. Autonomic cued reactivity in alcoholics: The effect of olfactory stimuli. Addict Behav. 1995;20(5):571–584. 8712055 10.1016/0306-4603(95)00017-7

[48] Fransson P, Marrelec G. The precuneus/posterior cingulate cortex plays a pivotal role in the default mode network: Evidence from a partial correlation network analysis. NeuroImage. 2008;42(3):1178–1184. 18598773 10.1016/j.neuroimage.2008.05.059

[49] Volkow DN, Wang G-J, Fowler SJ, Tomasi D. Addiction circuitry in the human brain. Annu Rev Pharmacol Toxicol. 2012;52(1):321–336. 21961707 10.1146/annurev-pharmtox-010611-134625 PMC3477468

[50] Fede JS, Kisner AM, Manuweera T, Kerich M, Momenan R. Compounding vulnerability in the neurocircuitry of addiction: Longitudinal functional connectivity changes in alcohol use disorder. Alcohol and Alcoholism. 2022;57:712–721. 35760068 10.1093/alcalc/agac028 PMC9651981

[51] Mogg K, Bradley PB. Anxiety and attention to threat: Cognitive mechanisms and treatment with attention bias modification. Behav Res Ther. 2016;87:76–108. 27616718 10.1016/j.brat.2016.08.001

[52] Bianco GM, Duggento A, Nigro S, Conti A, Toschi N, Passamonti L. Heritability of human “directed” functional connectome. Brain Behav. 2023;13(5):e2839. 36989125 10.1002/brb3.2839 PMC10175995

[53] Sullivan MR . Developmental changes in olfactory behavior and limbic circuitry. Chem Senses. 2005;30(Supplement 1):i152–i153. 15738086 10.1093/chemse/bjh159 PMC1868529

