# Supplementary material for: Anxiety modulates odour-linked brain connectivity and alcohol dependence risk
Source: Brain Commun. 2026 Mar 19;8(2):fcag096. doi: 10.1093/braincomms/fcag096 (PMC13023364; doi:10.1093/braincomms/fcag096)
Supplement: fcag096_Supplementary_Data [file fcag096_supplementary_data.docx]

**All codes**

**Code 1## Correlation between networks and odor scores**

# Load necessary libraries

library(tidyverse)

library(ppcor) # For partial correlation

# Load cleaned dataset

data_path <- "/Users/khushbuagarwal/Desktop/HCP_newproject/NBRC new study/merged_data_cleaned.csv"

df <- read.csv(data_path)

# Extract odor scores

odor_scores <- df$Odor_AgeAdj

# Identify connectivity columns correctly

connectivity_cols <- grep("^Connectivity_Node\\d+_Node\\d+$", names(df), value = TRUE)

# Select only the connectivity columns (already cleaned of duplicates)

brain_connections <- df[, connectivity_cols]

# Extract confounders

confounder_vars <- c("Age_in_Yrs", "Gender", "SSAGA_FTND_Score")

confounds <- df[, confounder_vars, drop = FALSE] # Keep it as a dataframe

# Convert categorical confounders (if necessary)

confounds$Gender <- as.numeric(confounds$Gender) # Ensure Gender is numeric

# Remove rows with missing values (IMPORTANT FIX)

complete_cases <- complete.cases(odor_scores, brain_connections, confounds)

brain_connections <- brain_connections[complete_cases, ]

odor_scores <- odor_scores[complete_cases]

confounds <- confounds[complete_cases, ]

# OPTIONAL: Split Data into Training and Validation Sets

set.seed(123) # For reproducibility

train_indices <- sample(1:nrow(brain_connections), size = 0.8 * nrow(brain_connections)) # 80% Training, 20% Validation

df_train <- brain_connections[train_indices, ]

df_test <- brain_connections[-train_indices, ]

# Pearson Correlation between Odor Scores and Connectivity

cor_results <- apply(brain_connections, 2, function(x) cor.test(x, odor_scores, method = "pearson"))

# Extract p-values and correlation coefficients

p_values <- sapply(cor_results, function(x) x$p.value)

cor_values <- sapply(cor_results, function(x) x$estimate)

# Bonferroni Correction for Multiple Comparisons

bonferroni_threshold <- 0.05 / length(p_values) # Adjusted significance threshold

significant_connections <- names(p_values[p_values < bonferroni_threshold])

# Partial Correlation Controlling for Age, Sex, and Smoking Status

partial_corr_results <- apply(brain_connections, 2, function(x) {

pcor.test(x, odor_scores, confounds)

})

# Extract p-values and partial correlation coefficients

partial_p_values <- sapply(partial_corr_results, function(x) x$p.value)

partial_cor_values <- sapply(partial_corr_results, function(x) x$estimate)

# Bonferroni Correction for Partial Correlations

partial_significant_connections <- names(partial_p_values[partial_p_values < bonferroni_threshold])

# Print Results

cat("Significant Pearson correlations (Bonferroni corrected):\n")

print(significant_connections)

cat("\nSignificant Partial correlations (Bonferroni corrected):\n")

print(partial_significant_connections)

# Define output directory

output_dir <- "/Users/khushbuagarwal/Desktop/HCP_newproject/NBRC_new_study"

# Ensure the output directory exists

if (!dir.exists(output_dir)) {

dir.create(output_dir, recursive = TRUE)

}

# Save results to CSV

output_file <- file.path(output_dir, "correlation_results_cleaned.csv")

results <- data.frame(

Connection = colnames(brain_connections),

Pearson_Correlation = cor_values,

Pearson_pValue = p_values,

Partial_Correlation = partial_cor_values,

Partial_pValue = partial_p_values

)

write.csv(results, output_file, row.names = FALSE)

cat("Results saved to:", output_file)

**Code 2 ## Stratified splitting**

library(dplyr)

library(caret) # for stratified split

# Load the dataset

file_path <- "/Users/khushbuagarwal/Desktop/HCP_newproject/NBRC new study/merged_data_cleaned.csv"

data <- read.csv(file_path, stringsAsFactors = FALSE)

# Set seed for reproducibility

set.seed(123)

# Ensure the target column is a factor (for stratification)

data$SSAGA_Alc_D4_Dp_Dx.x <- as.factor(data$SSAGA_Alc_D4_Dp_Dx.x)

# Perform stratified split (70% train, 30% validation)

split_index <- createDataPartition(data$SSAGA_Alc_D4_Dp_Dx.x, p = 0.7, list = FALSE)

train_data <- data[split_index, ]

validation_data <- data[-split_index, ]

# Save the datasets

train_file <- "/Users/khushbuagarwal/Desktop/HCP_newproject/NBRC new study/train_data_stratified.csv"

validation_file <- "/Users/khushbuagarwal/Desktop/HCP_newproject/NBRC new study/validation_data_stratified.csv"

write.csv(train_data, train_file, row.names = FALSE)

write.csv(validation_data, validation_file, row.names = FALSE)

# Print class distributions for sanity check

cat("Training Data:\n")

print(prop.table(table(train_data$SSAGA_Alc_D4_Dp_Dx.x)))

cat("Validation Data:\n")

print(prop.table(table(validation_data$SSAGA_Alc_D4_Dp_Dx.x)))

cat("\nStratified datasets saved successfully!\n")

**Code 3## top 10 features selection**

# Load required libraries

library(caret)

library(tidyverse)

library(doParallel)

library(ranger)

library(pROC)

library(smotefamily)

library(xgboost)

library(glmnet)

library(caretEnsemble)

library(ggplot2)

library(rmarkdown)

# File paths

train_path <- "/Users/khushbuagarwal/Desktop/HCP_newproject/NBRC new study/train_data_stratified.csv"

val_path <- "/Users/khushbuagarwal/Desktop/HCP_newproject/NBRC new study/validation_data_stratified.csv"

output_dir <- dirname(train_path)

output_file <- file.path(output_dir, "model_results_stratified_top10.csv")

# Read data

train_data <- read.csv(train_path)

validation_data <- read.csv(val_path)

# Create updated binary targets

make_binary_targets <- function(data) {

data %>%

mutate(

Risky_Drinks_7days = factor(ifelse(Total_Drinks_7days.x >= 14 | Num_Days_Drank_7days.x >= 4, "Yes", "No"), levels = c("No", "Yes")),

Risky_12Month = factor(ifelse(SSAGA_Alc_12_Drinks_Per_Day.x >= 4 | SSAGA_Alc_12_Frq.x >= 4, "Yes", "No"), levels = c("No", "Yes")),

SSAGA_Alc_D4_Dp_Dx.x = factor(SSAGA_Alc_D4_Dp_Dx.x, levels = c(1, 5), labels = c("No", "Yes"))

)

}

train_data <- make_binary_targets(train_data)

validation_data <- make_binary_targets(validation_data)

# Define connectivity features and targets

connectivity_features <- grep("^Connectivity_", names(train_data), value = TRUE)

alcohol_targets <- c("SSAGA_Alc_D4_Dp_Dx.x", "Risky_Drinks_7days", "Risky_12Month")

# Parallel setup

cores <- parallel::detectCores() - 1

cl <- makeCluster(cores)

registerDoParallel(cl)

# CV settings

cv_control <- trainControl(

method = "cv",

number = 3,

classProbs = TRUE,

summaryFunction = twoClassSummary,

sampling = "smote",

allowParallel = TRUE

)

# Store results

results <- data.frame()

for (target in alcohol_targets) {

cat("\n--- Processing:", target, "---\n")

df_model <- train_data %>% select(all_of(c(connectivity_features, target))) %>% na.omit()

val_model <- validation_data %>% select(all_of(c(connectivity_features, target))) %>% na.omit()

df_model[[target]] <- factor(df_model[[target]], levels = c("No", "Yes"))

val_model[[target]] <- factor(val_model[[target]], levels = c("No", "Yes"))

# Feature importance

set.seed(123)

rf_imp_model <- train(

x = df_model[, connectivity_features],

y = df_model[[target]],

method = "ranger",

trControl = trainControl(method = "none"),

importance = "impurity"

)

imp_df <- varImp(rf_imp_model)$importance %>%

rownames_to_column("Feature") %>%

arrange(desc(Overall)) %>%

slice_head(n = 10)

top_features <- imp_df$Feature

# Final dataset

df_final <- df_model %>% select(all_of(c(top_features, target)))

val_final <- val_model %>% select(all_of(c(top_features, target)))

if (length(unique(df_final[[target]])) < 2 || length(unique(val_final[[target]])) < 2) {

cat("Skipping due to class imbalance in", target, "\n")

next

}

# Train model

set.seed(42)

rf_model <- train(

as.formula(paste(target, "~ .")),

data = df_final,

method = "ranger",

trControl = cv_control,

metric = "ROC"

)

# Predict on validation

val_probs <- predict(rf_model, newdata = val_final, type = "prob")[, "Yes"]

val_pred <- factor(ifelse(val_probs > 0.5, "Yes", "No"), levels = c("No", "Yes"))

val_actual <- factor(val_final[[target]], levels = c("No", "Yes"))

# Ensure length match

if (length(val_pred) != length(val_actual)) {

cat("Skipping due to prediction length mismatch in", target, "\n")

next

}

# Confusion and ROC

conf_val <- caret::confusionMatrix(val_pred, val_actual, positive = "Yes")

roc_val <- tryCatch(pROC::roc(val_actual, val_probs), error = function(e) NA)

# Training performance

train_preds <- rf_model$pred

train_preds$pred <- factor(train_preds$pred, levels = c("No", "Yes"))

train_preds$obs <- factor(train_preds$obs, levels = c("No", "Yes"))

conf_train <- caret::confusionMatrix(train_preds$pred, train_preds$obs, positive = "Yes")

roc_train <- tryCatch(pROC::roc(train_preds$obs, train_preds$Yes), error = function(e) NA)

# Store performance

results <- rbind(results, data.frame(

Target = target,

Accuracy_Train = round(conf_train$overall["Accuracy"], 4),

Precision_Train = round(conf_train$byClass["Precision"], 4),

Recall_Train = round(conf_train$byClass["Recall"], 4),

F1_Train = round(conf_train$byClass["F1"], 4),

AUC_Train = ifelse(is.na(roc_train), NA, round(auc(roc_train), 4)),

Accuracy_Validation = round(conf_val$overall["Accuracy"], 4),

Precision_Validation = round(conf_val$byClass["Precision"], 4),

Recall_Validation = round(conf_val$byClass["Recall"], 4),

F1_Validation = round(conf_val$byClass["F1"], 4),

AUC_Validation = ifelse(is.na(roc_val), NA, round(auc(roc_val), 4))

))

# Save outputs

write.csv(data.frame(Actual = val_actual, Predicted = val_pred, Prob_Yes = val_probs),

file.path(output_dir, paste0("predictions_", target, ".csv")), row.names = FALSE)

write.csv(imp_df, file.path(output_dir, paste0("feature_importance_", target, ".csv")), row.names = FALSE)

}

# Final result write

write.csv(results, output_file, row.names = FALSE)

# Clean up

stopCluster(cl)

cat("\u2705 All models completed and results saved to:", output_file, "\n")

**Code 4## Validation accuracy top 10 features**

# Load required libraries

library(caret)

library(tidyverse)

library(doParallel)

library(ranger)

library(pROC)

library(smotefamily)

library(xgboost)

library(glmnet)

library(caretEnsemble)

library(ggplot2)

library(rmarkdown)

# File paths

train_path <- "/Users/khushbuagarwal/Desktop/HCP_newproject/NBRC new study/train_data_stratified.csv"

val_path <- "/Users/khushbuagarwal/Desktop/HCP_newproject/NBRC new study/validation_data_stratified.csv"

output_dir <- dirname(train_path)

output_file <- file.path(output_dir, "model_results_stratified_top10.csv")

report_rmd <- file.path(output_dir, "model_summary_report.Rmd")

# Read data

df <- read.csv(train_path)

validation_data <- read.csv(val_path)

# -------- Create Derived Binary Targets --------

make_binary_targets <- function(data) {

data <- data %>%

mutate(

Risky_Drinks_7days = ifelse(Total_Drinks_7days.x >= 14 | Num_Days_Drank_7days.x >= 4, "Yes", "No"),

Risky_12Month = ifelse(SSAGA_Alc_12_Drinks_Per_Day.x >= 4 | SSAGA_Alc_12_Frq.x >= 4, "Yes", "No"),

SSAGA_Alc_D4_Dp_Dx.x = factor(SSAGA_Alc_D4_Dp_Dx.x, levels = c(1, 5), labels = c("No", "Yes")),

Risky_Drinks_7days = factor(Risky_Drinks_7days, levels = c("No", "Yes")),

Risky_12Month = factor(Risky_12Month, levels = c("No", "Yes"))

)

return(data)

}

df <- make_binary_targets(df)

validation_data <- make_binary_targets(validation_data)

# Connectivity features

connectivity_features <- grep("^Connectivity_", names(df), value = TRUE)

# Classification targets

classification_targets <- c("SSAGA_Alc_D4_Dp_Dx.x", "Risky_Drinks_7days", "Risky_12Month")

# Parallel backend

cores <- parallel::detectCores() - 1

cl <- makeCluster(cores)

registerDoParallel(cl)

# CV control

cv_control <- trainControl(

method = "cv",

number = 10,

classProbs = TRUE,

summaryFunction = twoClassSummary,

savePredictions = "final",

sampling = "smote",

allowParallel = TRUE

)

# Results container

results <- list()

for (target in classification_targets) {

cat("\n--- Target:", target, "---\n")

df_model <- df[, c(connectivity_features, target)] %>% na.omit()

val_model <- validation_data[, c(connectivity_features, target)] %>% na.omit()

X <- df_model[, connectivity_features]

Y <- df_model[[target]]

# Ensure factor consistency

Y <- factor(Y, levels = c("No", "Yes"))

df_model[[target]] <- Y

val_model[[target]] <- factor(val_model[[target]], levels = c("No", "Yes"))

# Show class balance

cat("Train Class Balance:\n"); print(table(Y))

cat("Validation Class Balance:\n"); print(table(val_model[[target]]))

# Feature selection via Random Forest

rf_feat_model <- train(

x = X, y = Y,

method = "ranger",

trControl = cv_control,

metric = "ROC",

importance = "impurity"

)

var_imp <- varImp(rf_feat_model)$importance %>% rownames_to_column("Feature")

top10_df <- var_imp %>% arrange(desc(Overall)) %>% head(10)

top10_feats <- top10_df$Feature

# Save feature importance and plot

write.csv(var_imp, file.path(output_dir, paste0("importance_", target, ".csv")), row.names = FALSE)

plot <- ggplot(top10_df, aes(x = reorder(Feature, Overall), y = Overall)) +

geom_col(fill = "steelblue") +

coord_flip() +

ggtitle(paste("Feature Importance -", target)) +

theme_minimal()

ggsave(file.path(output_dir, paste0("feature_plot_", target, ".png")), plot = plot)

# Subset for top 10 features

df_subset <- df_model[, c(top10_feats, target)]

val_subset <- val_model[, c(top10_feats, target)]

# Ensemble Models

model_list <- caretList(

as.formula(paste(target, "~ .")),

data = df_subset,

trControl = cv_control,

metric = "ROC",

tuneList = list(

rf = caretModelSpec(method = "ranger"),

xgb = caretModelSpec(method = "xgbTree"),

glmnet = caretModelSpec(method = "glmnet")

)

)

# Stacked model

ens_model <- caretStack(model_list, method = "glm", metric = "ROC", trControl = cv_control)

# Predict on validation set

probs <- predict(ens_model, newdata = val_subset)

val_actual <- val_subset[[target]]

val_pred <- factor(ifelse(probs > 0.5, "Yes", "No"), levels = c("No", "Yes"))

conf <- confusionMatrix(val_pred, val_actual, positive = "Yes")

auc_val <- auc(roc(val_actual, probs))

# Save ROC Curve

roc_plot <- ggroc(roc(val_actual, probs)) + ggtitle(paste("ROC -", target)) + theme_minimal()

ggsave(file.path(output_dir, paste0("roc_plot_", target, ".png")), plot = roc_plot)

# Save predictions

pred_df <- data.frame(ID = 1:nrow(val_subset), Actual = val_actual, Predicted = val_pred, Prob = probs)

write.csv(pred_df, file.path(output_dir, paste0("predictions_", target, ".csv")), row.names = FALSE)

# Store results

results[[target]] <- list(

Accuracy = conf$overall["Accuracy"],

Precision = ifelse(is.na(conf$byClass["Precision"]), 0, conf$byClass["Precision"]),

Recall = ifelse(is.na(conf$byClass["Recall"]), 0, conf$byClass["Recall"]),

F1 = ifelse(is.na(conf$byClass["F1"]), 0, conf$byClass["F1"]),

AUC = auc_val,

Features = top10_feats,

EnsembleModel = ens_model

)

}

# Save model performance summary

overall_df <- map_dfr(names(results), function(name) {

res <- results[[name]]

data.frame(

Target = name,

Accuracy = round(res$Accuracy, 4),

Precision = round(res$Precision, 4),

Recall = round(res$Recall, 4),

F1 = round(res$F1, 4),

AUC = round(res$AUC, 4),

Num_Features = length(res$Features),

stringsAsFactors = FALSE

)

})

write.csv(overall_df, output_file, row.names = FALSE)

# Optional PDF Report

if (!file.exists(report_rmd)) {

writeLines(c(

"---",

"title: 'Model Summary Report'",

"output: pdf_document",

"---",

"",

"```{r load, include=FALSE}",

"library(ggplot2)",

"library(readr)",

paste0("results <- read.csv(\"", output_file, "\")"),

"```",

"",

"```{r summary, echo=FALSE}",

"knitr::kable(results)",

"```"

), con = report_rmd)

}

rmarkdown::render(report_rmd)

# Clean up

stopCluster(cl)

cat("\u2705 Modeling completed. Results and report saved to:", output_dir, "\n")

**Code 5## Create alcohol binaries and Validation Modelling**

# ===== Load Required Libraries =====

library(caret)

library(tidyverse)

library(pROC)

library(ranger)

library(xgboost)

library(glmnet)

library(doParallel)

library(ggplot2)

library(smotefamily)

library(reshape2)

library(boot)

library(iml)

library(knitr)

library(ggpubr)

library(RColorBrewer)

library(kableExtra)

library(rmarkdown)

# ===== Set Seed for Reproducibility =====

set.seed(42)

# ===== File Paths =====

train_path <- "/Users/khushbuagarwal/Desktop/HCP_newproject/NBRC new study/train_data_with_anxiety_vars.csv"

val_path <- "/Users/khushbuagarwal/Desktop/HCP_newproject/NBRC new study/validation_data_with_anxiety_vars.csv"

output_dir <- dirname(train_path)

dir.create(output_dir, showWarnings = FALSE)

# ===== Load Data =====

df <- read.csv(train_path)

validation_data <- read.csv(val_path)

# ===== Create Derived Binary Targets =====

make_binary_targets <- function(data) {

data %>%

mutate(

Risky_Drinks_7days = factor(ifelse(Total_Drinks_7days.x >= 14 | Num_Days_Drank_7days.x >= 4, "Yes", "No"), levels = c("No", "Yes")),

Risky_12Month = factor(ifelse(SSAGA_Alc_12_Drinks_Per_Day.x >= 4 | SSAGA_Alc_12_Frq.x >= 4, "Yes", "No"), levels = c("No", "Yes")),

SSAGA_Alc_Dp.x = factor(ifelse(SSAGA_Alc_Dp.x == 5, "Yes", "No"), levels = c("No", "Yes"))

)

}

df <- make_binary_targets(df)

validation_data <- make_binary_targets(validation_data)

# ===== Define Features =====

odor_features <- c(

"Connectivity_Node15_Node1", "Connectivity_Node12_Node2", "Connectivity_Node18_Node2",

"Connectivity_Node21_Node2", "Connectivity_Node31_Node5", "Connectivity_Node44_Node5",

"Connectivity_Node13_Node6", "Connectivity_Node40_Node6", "Connectivity_Node13_Node8",

"Connectivity_Node21_Node9", "Connectivity_Node31_Node9", "Connectivity_Node40_Node9",

"Connectivity_Node21_Node12", "Connectivity_Node27_Node14", "Connectivity_Node25_Node15",

"Connectivity_Node27_Node15", "Connectivity_Node40_Node15", "Connectivity_Node24_Node17",

"Connectivity_Node31_Node18", "Connectivity_Node32_Node18", "Connectivity_Node40_Node18",

"Connectivity_Node22_Node19", "Connectivity_Node24_Node19", "Connectivity_Node22_Node21",

"Connectivity_Node38_Node21", "Connectivity_Node30_Node24", "Connectivity_Node40_Node34"

)

features_with_anxiety <- c(odor_features, "DSM_Anxi_T")

features_without_anxiety <- odor_features

feature_sets <- list(With_Anxiety = features_with_anxiety, Without_Anxiety = features_without_anxiety)

classification_targets <- c("SSAGA_Alc_Dp.x", "Risky_Drinks_7days", "Risky_12Month")

models_to_use <- c("ranger", "xgbTree", "glmnet")

# ===== Parallel Setup =====

cores <- min(4, detectCores() - 1)

cl <- makeCluster(cores)

registerDoParallel(cl)

on.exit(stopCluster(cl), add = TRUE)

# ===== Train Control Settings =====

cv_control <- trainControl(

method = "cv",

number = 5,

classProbs = TRUE,

summaryFunction = twoClassSummary,

savePredictions = "final",

sampling = "smote",

allowParallel = TRUE

)

# ===== Initialize Results =====

results <- list()

# ===== Helper: Check both classes =====

has_both_classes <- function(x) {

all(c("No", "Yes") %in% unique(x))

}

# ===== Loop Through Feature Sets and Targets =====

for (feat_set_name in names(feature_sets)) {

features <- feature_sets[[feat_set_name]]

for (target in classification_targets) {

cat("\nFeature Set:", feat_set_name, "| Target:", target, "\n")

if (!all(features %in% colnames(df))) {

warning("Missing features in dataset for ", feat_set_name)

next

}

df_model <- na.omit(df[, c(features, target)])

val_model <- na.omit(validation_data[, c(features, target)])

if (nrow(df_model) < 30 || nrow(val_model) < 10) {

warning("Skipping due to insufficient data.")

next

}

df_model[[target]] <- factor(df_model[[target]], levels = c("No", "Yes"))

val_model[[target]] <- factor(val_model[[target]], levels = c("No", "Yes"))

model_metrics <- list()

model_probs <- list()

for (model_name in models_to_use) {

cat(" - Training model:", model_name, "\n")

model <- tryCatch({

train(as.formula(paste(target, "~ .")), data = df_model, method = model_name, trControl = cv_control, metric = "ROC")

}, error = function(e) {

warning("Training failed for", model_name, ":", e$message)

return(NULL)

})

if (is.null(model)) next

val_probs <- predict(model, newdata = val_model, type = "prob")[, "Yes"]

val_actual <- val_model[[target]]

val_pred <- factor(ifelse(val_probs > 0.5, "Yes", "No"), levels = c("No", "Yes"))

roc_obj <- tryCatch({

roc(val_actual, val_probs, levels = c("No", "Yes"), direction = "<")

}, error = function(e) NULL)

if (!is.null(roc_obj)) {

ggsave(file.path(output_dir, paste0("roc_", feat_set_name, "_", target, "_", model_name, ".png")),

ggroc(roc_obj) + ggtitle(paste("ROC -", feat_set_name, target, model_name)) + theme_minimal(),

width = 6, height = 4)

}

conf <- confusionMatrix(val_pred, val_actual, positive = "Yes")

model_metrics[[model_name]] <- list(

Accuracy = conf$overall["Accuracy"],

Precision = conf$byClass["Precision"],

Recall = conf$byClass["Recall"],

F1 = conf$byClass["F1"],

AUC = if (!is.null(roc_obj)) auc(roc_obj) else NA

)

model_probs[[model_name]] <- val_probs

if (model_name == "ranger" && target == "Risky_12Month") {

tryCatch({

predictor <- Predictor$new(model, data = df_model[, features], y = df_model[[target]], type = "prob")

shap <- Shapley$new(predictor, x.interest = df_model[1, features])

png(file.path(output_dir, paste0("shap_", feat_set_name, "_", target, ".png")))

plot(shap)

dev.off()

}, error = function(e) message("SHAP failed: ", e$message))

}

}

if (length(model_probs) == 0) next

soft_probs <- rowMeans(as.data.frame(model_probs))

actual <- val_model[[target]]

soft_pred <- factor(ifelse(soft_probs > 0.5, "Yes", "No"), levels = c("No", "Yes"))

conf_soft <- confusionMatrix(soft_pred, actual, positive = "Yes")

if (has_both_classes(actual)) {

roc_soft <- roc(actual, soft_probs, levels = c("No", "Yes"), direction = "<")

auc_soft <- auc(roc_soft)

} else {

warning("Soft voting: Only one class in actual. Skipping AUC.")

auc_soft <- NA

}

hard_preds_df <- as.data.frame(lapply(model_probs, function(p) factor(ifelse(p > 0.5, "Yes", "No"), levels = c("No", "Yes"))))

hard_vote <- apply(hard_preds_df, 1, function(row) names(which.max(table(row))))

hard_pred <- factor(hard_vote, levels = c("No", "Yes"))

conf_hard <- confusionMatrix(hard_pred, actual, positive = "Yes")

if (has_both_classes(actual)) {

roc_hard <- roc(actual, as.numeric(hard_pred == "Yes"), levels = c(0, 1), direction = "<")

auc_hard <- auc(roc_hard)

} else {

warning("Hard voting: Only one class in actual. Skipping AUC.")

auc_hard <- NA

}

for (model_name in names(model_metrics)) {

m <- model_metrics[[model_name]]

results[[length(results) + 1]] <- data.frame(

Feature_Set = feat_set_name,

Target = target,

Model = model_name,

Accuracy = round(m$Accuracy, 4),

Precision = round(m$Precision, 4),

Recall = round(m$Recall, 4),

F1 = round(m$F1, 4),

AUC = round(m$AUC, 4)

)

}

results[[length(results) + 1]] <- data.frame(

Feature_Set = feat_set_name,

Target = target,

Model = "Soft_Vote",

Accuracy = round(conf_soft$overall["Accuracy"], 4),

Precision = round(conf_soft$byClass["Precision"], 4),

Recall = round(conf_soft$byClass["Recall"], 4),

F1 = round(conf_soft$byClass["F1"], 4),

AUC = round(auc_soft, 4)

)

results[[length(results) + 1]] <- data.frame(

Feature_Set = feat_set_name,

Target = target,

Model = "Hard_Vote",

Accuracy = round(conf_hard$overall["Accuracy"], 4),

Precision = round(conf_hard$byClass["Precision"], 4),

Recall = round(conf_hard$byClass["Recall"], 4),

F1 = round(conf_hard$byClass["F1"], 4),

AUC = round(auc_hard, 4)

)

}

}

# ===== Export Results =====

summary_df <- bind_rows(results)

write.csv(summary_df, file.path(output_dir, "model_summary.csv"), row.names = FALSE)

cat("\n Summary saved to:", file.path(output_dir, "model_summary.csv"), "\n")

**Supplemental Table S1 |** Post hoc regression model results for alcohol dependence

|  | ***Alcohol Dependence/ElasticNet/Training*** | | |
| --- | --- | --- | --- |
| **Features** | **Odds Ratio** | **CI** | **P value** |
| Connectivity_Node15_Node2 | 3.13 | 0.70 – 18.19 | 0.15 |
| Connectivity_Node37_Node12 | 1.01 | 0.96 – 1.00 | 0.01 |
| Connectivity_Node15_Node8 | 1.12 | 0.92 – 0.97 | <0.001 |
| Connectivity_Node25_Node15 | 2.34 | 0.74 – 8.03 | 0.14 |
| Connectivity_Node30_Node24 | 0.003 | 0.0001 – 0.03 | <0.001 |
| Connectivity_Node21_Node12 | 0.02 | 0.00 – 0.28 | 0.003 |
| Connectivity_Node26_Node7 | 0.16 | 0.02 – 1.01 | 0.05 |
| Connectivity_Node14_Node11 | 0.10 | 0.01 – 0.82 | 0.05 |
| Connectivity_Node12_Node8 | 4.27 | 0.36 – 36.79 | 0.19 |
| Connectivity_Node44_Node11 | 3.57 | 0.60 – 19.33 | 0.13 |
| Anxiety | 3.01 | 1.53 – 6.51 | **0.001** |
| Connectivity_Node15_Node2*Anxiety | 0.97 | 0.94 – 1.01 | 0.12 |
| Connectivity_Node37_Node12*Anxiety | 0.86 | 0.81 – 0.91 | **<0.001** |
| Connectivity_Node15_Node8*Anxiety | 0.80 | 0.73 – 0.86 | **<0.001** |
| Connectivity_Node25_Node15*Anxiety | 0.98 | 0.95 – 1.0 | 0.06 |
| Connectivity_Node30_Node24*Anxiety | 1.11 | 1.06 – 1.17 | **<0.001** |
| Connectivity_Node21_Node12*Anxiety | 1.06 | 1.01 – 1.11 | **0.01** |
| Connectivity_Node26_Node7*Anxiety | 1.03 | 0.99 – 1.06 | 0.08 |
| Connectivity_Node14_Node11*Anxiety | 1.04 | 1.00 – 1.1 | **0.04** |

| Connectivity_Node12_Node8*Anxiety | 0.97 | 0.93 – 1.02 | 0.24 |
| --- | --- | --- | --- |
| Connectivity_Node44_Node11*Anxiety | 1.00 | 1.00 – 1.00 | **0.02** |
|  | ***Alcohol Dependence/LASSO/Training*** | | |
| Connectivity_Node15_Node2 | 1.01 | 0.99 – 1.02 | 0.31 |
| Connectivity_Node37_Node12 | 0.97 | 0.97 – 0.95 | 0.00 |
| Connectivity_Node15_Node8 | 1.01 | 0.91 – 0.96 | <0.001 |
| Connectivity_Node25_Node15 | 0.93 | 0.91 – 0.95 | <0.001 |
| Connectivity_Node30_Node24 | 0.001 | 0.00 – 0.02 | <0.001 |
| Connectivity_Node21_Node12 | 0.01 | 0.0 – 0.14 | 0.001 |
| Connectivity_Node26_Node7 | 0.06 | 0.01 – 0.28 | 0.003 |
| Connectivity_Node14_Node11 | 0.16 | 0.01 – 1.25 | 0.10 |
| Connectivity_Node12_Node8 | 1.17 | 1.02 – 1.35 | 0.02 |
| Connectivity_Node44_Node11 | 3.57 | 0.6 – 19.33 | 0.13 |
| Anxiety | 2.66 | 1.42 – 5.18 | 0.002 |
| Connectivity_Node15_Node2*Anxiety | 0.99 | 0.99 – 0.99 | 0.05 |
| Connectivity_Node37_Node12*Anxiety | 0.85 | 0.79 – 0.90 | **<0.001** |
| Connectivity_Node15_Node8*Anxiety | 0.81 | 0.75 – 0.86 | **<0.001** |
| Connectivity_Node25_Node15*Anxiety | 0.99 | 0.99 – 0.99 | **<0.001** |
| Connectivity_Node30_Node24*Anxiety | 1.13 | 1.07 – 1.20 | **<0.001** |
| Connectivity_Node21_Node12*Anxiety | 1.08 | 1.02 – 1.14 | **0.003** |
| Connectivity_Node26_Node7*Anxiety | 1.04 | 1.02 – 1.08 | **0.01** |
| Connectivity_Node14_Node11*Anxiety | 1.03 | 0.99 – 1.08 | 0.09 |
| Connectivity_Node44_Node11*Anxiety | 0.97 | 0.94 – 1.01 | 0.16 |

**Note:** Here, Connectivity_Node15_Node2 = Node 15: precuneus cortex, cingulate gyrus, left accumbens; Node 2: angular gyrus, middle temporal gyrus - posterior part, accumbens, cerebral cortex; Connectivity_Node37_Node12 = Node37: inferior frontal gyrus, pars opercularis; Node 12: angular gyrus, supramarginal gyrus, cerebral cortex; Connectivity_Node15_Node8 = Node 15: precuneus cortex, cingulate gyrus, left accumbens; Node 8: lateral occipital cortex, left

hippocampus; Connectivity_Node25_Node15 = Node 25: occipital pole, right amygdala; Node 15: precuneus cortex, cingulate gyrus, left accumbens; Connectivity_Node30_Node24 = Node 30: lateral occipital cortex, left amygdala; Node 24: inferior temporal gyrus, left amygdala; Connectivity_Node21_Node12 = Node 21: postcentral gyrus, precentral gyrus, cerebral cortex; Node 12: angular gyrus, supramarginal gyrus, cerebral cortex; Connectivity_Node26_Node7 = Node 26: frontal operculum cortex, paracingulate gyrus; Node 7: supramarginal gyrus; Connectivity_Node14_Node11 = Node 14: inferior temporal gyrus, left caudate; Node 11: occipital fusiform gyrus, lingual gyrus; Connectivity_Node12_Node8 = Node 12: angular gyrus, supramarginal gyrus, cerebral cortex; Node 8: lateral occipital cortex, left hippocampus; Connectivity_Node44_Node11 = Node 44: middle temporal gyrus, inferior frontal gyrus, left accumbens; Node 11: occipital fusiform gyrus, lingual gyrus.

**Supplemental Table S2 |** Post hoc regression model results for past 12 months risky drinking

|  | ***Past 12 months risky drinking/ElasticNet/Training*** | | |
| --- | --- | --- | --- |
| **Features** | **Odds Ratio** | **CI** | **P value** |
| Connectivity_Node15_Node2 | 1.05 | 1.0 – 1.10 | 0.03 |
| Connectivity_Node37_Node12 | 0.57 | 0.30 – 1.05 | 0.08 |
| Connectivity_Node15_Node8 | 1.54 | 0.67 – 3.89 | 0.32 |
| Connectivity_Node25_Node15 | 0.93 | 0.87 – 0.98 | 0.01 |
| Connectivity_Node30_Node24 | 0.49 | 0.20 – 1.10 | 0.09 |
| Connectivity_Node21_Node12 | 0.62 | 0.21 – 1.65 | 0.35 |
| Connectivity_Node26_Node7 | 0.43 | 0.24 – 0.75 | 0.003 |
| Connectivity_Node14_Node11 | 0.93 | 0.88 – 0.98 | 0.01 |
| Anxiety | 0.61 | 0.44 – 0.82 | 0.001 |
| Connectivity_Node37_Node12*Anxiety | 1.01 | 1.0 – 1.02 | **0.04** |
| Connectivity_Node15_Node8*Anxiety | 0.99 | 0.90 – 1.00 | 0.23 |
| Connectivity_Node30_Node24*Anxiety | 1.01 | 1.0 – 1.03 | 0.05 |
| Connectivity_Node21_Node12*Anxiety | 1.00 | 0.98 – 1.02 | 0.41 |
| Connectivity_Node26_Node7*Anxiety | 1.01 | 1.0 – 1.02 | **0.002** |
|  | ***Past 12 months risky drinking/LASSO/Training*** | | |
| Connectivity_Node37_Node12 | 1.04 | 1.00 – 1.08 | 0.01 |
| Connectivity_Node15_Node8 | 0.97 | 0.95 – 1.00 | 0.08 |
| Anxiety | 2.66 | 1.42 – 5.18 | 0.002 |
| Connectivity_Node15_Node8*Anxiety | 0.99 | 0.99 – 0.99 | **0.004** |

**Note:** Here, Connectivity_Node15_Node2 = Node 15: precuneus cortex, cingulate gyrus, left accumbens; Node 2: angular gyrus, middle temporal gyrus - posterior part, accumbens, cerebral cortex; Connectivity_Node37_Node12 = Node37: inferior frontal gyrus, pars opercularis; Node 12: angular gyrus, supramarginal gyrus, cerebral cortex; Connectivity_Node15_Node8 = Node 15: precuneus cortex, cingulate gyrus, left accumbens; Node 8: lateral occipital cortex, left

hippocampus; Connectivity_Node25_Node15 = Node 25: occipital pole, right amygdala; Node 15: precuneus cortex, cingulate gyrus, left accumbens; Connectivity_Node30_Node24 = Node 30: lateral occipital cortex, left amygdala; Node 24: inferior temporal gyrus, left amygdala; Connectivity_Node21_Node12 = Node 21: postcentral gyrus, precentral gyrus, cerebral cortex; Node 12: angular gyrus, supramarginal gyrus, cerebral cortex; Connectivity_Node26_Node7 = Node 26: frontal operculum cortex, paracingulate gyrus; Node 7: supramarginal gyrus; Connectivity_Node14_Node11 = Node 14: inferior temporal gyrus, left caudate; Node 11: occipital fusiform gyrus, lingual gyrus
